# A novel hairless highly immunodeficient mice model optimized for in vivo imaging

**DOI:** 10.1186/s42826-026-00275-9

**Published:** 2026-04-07

**Authors:** Kouki Matsuda, Ryusho Kariya, Sawako Fujikawa, Kenji Maeda, Seiji Okada

**Affiliations:** 1 Division of Hematopoiesis, Joint Research Center for Human Retrovirus Infection, Kumamoto University, 2-2-1 Honjo, Chuo-ku, Kumamoto, 860-0811 Japan; 2 Division of Antiviral Therapy, Joint Research Center for Human Retrovirus Infection, Kagoshima University, 8-35-1 Sakuragaoka, Kagoshima, 890-8544 Japan

**Keywords:** In vivo imaging, Immunodeficiency, Hairless mice, Tumor engraftment

## Abstract

**Background:**

In vivo imaging is one of the analytical technologies that has been rapidly growing in demand in recent years to non-invasively observe the behavior of molecules such as genes and proteins in vivo and perform quantitative and qualitative analysis. Since the hair blocks and absorbs the fluorescence, Nude and Hairless mice have been used for in vivo imaging. Nude mice have been used for in vivo imaging for a long time; however, more efficient mice are needed for the next generation of imaging.

**Results:**

We established a novel Hairless Rag2/Jak3 KO mice (Hairless R/J mice) model that exhibits hairlessness and a lack of T, B, and NK cell phenotypes. Hairless R/J mice exhibit thinner skin than that of Nude R/J mice. These mice also showed superior optical properties compared with Nude R/J mice, as demonstrated by green fluorescent beads and the mCherry-expressing human cholangiocarcinoma cell line M213, following subcutaneous transplantation. Furthermore, we show that Ihara cells, a human malignant melanoma cell line, can be used to facilitate live imaging of the growth of transplanted tumors.

**Conclusions:**

This novel mouse model will be a valuable tool for noninvasive tumor monitoring and evaluation of anticancer therapies.

**Supplementary Information:**

The online version contains supplementary material available at 10.1186/s42826-026-00275-9.

## Background

Preclinical animal models play a crucial role in cancer research, particularly in assessing tumor progression and therapeutic efficacy. Among these, immunodeficient mouse models are indispensable for xenograft studies using human cancer cells [1–3]. The commonly used nude mice (Foxn1^nu/nu^) lack T cells but retain other immune components, which may interfere with engrafted tumor growth and experimental outcomes [4–6]. More severely immunodeficient models, such as Rag2/Jak3 KO mice, provide a more suitable platform for human tumor xenografts by lacking both T and B cells, with additional impairments in NK cell function [7]. In our previous study, we established highly immunodeficient mice exhibiting Rag2/Jak3 KO with a nude mouse backbone [8]. While nude mice have facilitated numerous in vivo imaging studies, their residual hair follicles contribute to skin thickness, reducing the penetration of optical signals.

The Hairless (Hr) mouse was first described in 1954 as a spontaneous mutant lacking normal hair follicle development due to mutations in the Hr gene [9]. Previously, Schaffer et al. evaluated the immune function of hairless mice of the SKH1 strain and reported that they showed immune cell composition and tumor rejection comparable to C57BL/6 mice, making them a more physiologically accurate preclinical mouse model of immune-competent hairless [10]. Over the years, hairless mice have been widely used in dermatological research, especially in studies on skin barrier function, wound healing, and alopecia-related conditions [11, 12]. Their thin skin has also made them valuable in phototherapy studies and transdermal drug delivery research [13]. However, despite their advantages as alternative models for improving imaging clarity, immunodeficient hairless mouse models remain limited, and their potential use in cancer research has not been fully explored.

In this study, we established an immunodeficient hairless mouse model by introducing a Rag2/Jak3 knockout mutation, providing a useful tumor imaging platform. Since these mice have thinner skin compared with Nude mice, it can be a more useful model for human tumor cell engraftment and in vivo imaging.

## Methods

### Mice

BALB/c Hairless mice (ID nbio003) were obtained from Laboratory Animal Resource Bank, National Institutes of Biomedical Innovation, Health and Nutrition, Osaka, Japan. BALB/c Rag-2/Jak3 double-deficient (Rag2^-/-^Jak3^-/-^) (BRJ) mice were established by crossing BALB/c Rag2^-/-^ mice with BALB/c Jak3^-/-^ mice [7]. BALB/c Hairless Rag2^-/-^ Jak3^-/-^ (Hairless R/J) mice were established by crossing BALB/c Rag2^-/-^ Jak3^-/-^ mice with BALB/c Hairless mice. BALB/c Nude Rag-2/Jak3 double-deficient (Rag2^-/-^Jak3^-/-^) (Nude R/J) mice were established by crossing BRJ mice with BALB/c nude mice [8]. The Hairless [9], Rag-2, and Jak3 mutations were genotyped by PCR using genomic DNA extracted from tail tissue, as previously described. PCR genotyping for the *Hr* mutation was done using primers am05 (viral LTR) 5′-GCGTTACTGCAGCTAGCTTG-3′, am06 (*Hr* exon 6) 5′-TGTAGCCTGTGGTCGCATAG-3′, and am07 (*Hr* intron 6) 5′-CTCCTGTTTGCTTGGTCATC-3′, which produce a 350 bp product for the wild-type allele and a 250 bp product for the *SKH1* mutant allele. The Institutional Animal Care and Use Committee of Kumamoto University approved all experimental procedures and protocols　(A2021-053, A2023-153).

### Tumor cell preparation

The human cholangiocarcinoma cell line KKU-213A [10] and highly pigmented human melanoma cell line Ihara [11, 12] were cultured in Dulbecco’s Modified Eagle’s Medium (DMEM) (Wako Pure Chemicals, Osaka, Japan) supplemented with 10% (v/v) heat-inactivated fetal bovine serum (FBS; JRH Bioscience, Lenexa, KS, USA), 100 U/ml penicillin, 100 μg/ml streptomycin. The mCherry-transduced KKU-213A (M213-mCherry) was established by transfection of pmCherry-N1 Vector (Clontech, Mountain View, CA, USA) with the transfection reagent Lipofectamine 2000 (Invitrogen, Carlsbad, CA, USA) according to the manufacturer’s instructions, and selected in medium containing neomycin (G418; Carbiochem, Darmstadt, Germany), followed by isolation of stable clones by limiting dilution.

### Flow cytometry

Mouse splenocytes were isolated and stained with DX5-FITC (pan-NK marker), mCD122 (IL-2 Rβ)-PE, mCD19-APC, and mCD3-PE/Cy7 (eBiosciences, San Diego, CA, USA) and analyzed with LSR II (BD Biosciences, San Diego, CA, USA). Data were analyzed with FlowJo software (Tree Star, San Carlos, CA, USA).

### Measurement of skin thickness

Skin thickness was directly measured using an electric caliper (Mitsumoto, Kawasaki, Japan) at two locations per mouse, on the left and right sides. The thickness of the skin between Hairless R/J and Nude R/J mice (8–10-week-old females; 7 and 11 mice, respectively) was compared. The thickness of the epidermal portion was compared in HE-stained mouse skin tissues, measured at three locations per sample (Ten 8–10-week-old female mice per group).

### Skin administration of fluorescent beads in Nude R/J mice and Hairless R/J mice

Nude R/J mice and Hairless R/J mice (Seven 8–10 weeks old females) were administered subcutaneously in the back with 100 μl of Fluoresbrite YG Microspheres (Polyscience, Washington, PA). Fluorescence intensity was measured using the Maestro in vivo fluorescence imaging system (Cambridge Research & Instrumentation, Massachusetts, USA), and Fluorescence signals were quantified as total signal within the ROI and expressed in arbitrary units (a.u.).

### Tumor transplantation and measurement of tumor red fluorescence

Eight to ten weeks old female Nude R/J mice and Hairless R/J mice were inoculated subcutaneously in both flanks with 5 × 10^6^ KKU-213A-mCherry suspended in 100 μl phosphate-buffered saline (PBS). On day 16, xenograft mice were anesthetized, and fluorescence intensity was measured using the Maestro. Then, they were sacrificed, and tumors were removed and weighed. The fluorescence intensity of the removed tumors was also measured. Ihara cells (1 × 10^6^ /mouse) were transplanted subcutaneously in both flanks of Hairless R/J mice. On day 21, xenograft mice were sacrificed, tumors were removed, and histological analysis was performed to confirm tumor engraftment.

### Histological analysis

Skin from the mice and tumors were fixed in 10% neutral-buffered formalin immediately after removal, embedded in paraffin, sectioned at 4 µm, and stained with hematoxylin-eosin (H&E). Xenografted Ihara cells were prepared into tissue sections from the tumor-containing area and skin, and melanoma engraftment was confirmed by Masson-Fontana staining.

### Calculation of tumor volume

Tumor size derived from xenografted KKU-213A-mCherry cells was measured by electric calipers, and tumor volume (V) was calculated as below: V = L×W2 × 0.52, where *L* and *W* were the longest and shortest tumor dimensions, respectively [13].

### Statistical analysis

The statistical significance of differences observed between experimental groups was determined using an Unpaired t-test. Data are presented as mean with SD. The correlation analysis of tumor intensity, volume, and weight was assessed with Pearson’s r. *p*-values less than 0.05 were considered significant. All analyses were performed using GraphPad Prism version 8 (GraphPad Software, La Jolla, CA).

## Results

### Hairless Rag2/Jak3 KO (Hairless R/J) mice exhibit a complete loss of lymphocytes and NK cells

**Fig. 1:**
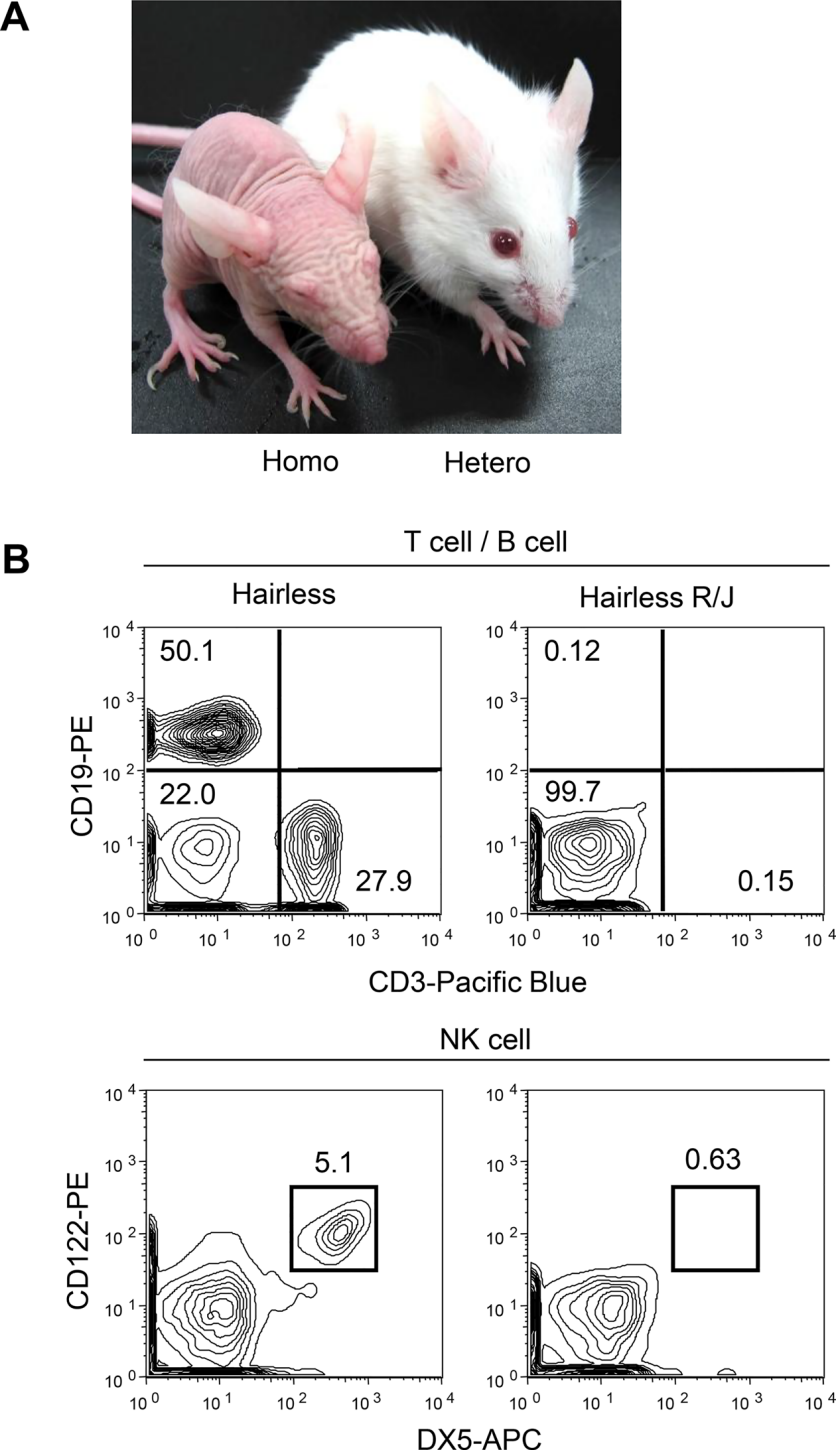
Characteristic of hairless R/J mice. (**A**) The appearance of hairless mice (left: homo, right: hetero). (**B**) Flow cytometric analysis of lineage markers of the Hairless mice and Hairless R/J mice spleen cells. Lack of mature B and T lymphocytes and NK cells in Hairless R/J mice. Spleen cells from Hairless wild-type mice and Hairless R/J mice (6 weeks-old female) were stained with anti-mouse CD19-PE and CD3-Pacific Blue, or DX5-APC (pan NK marker) and anti-mouse CD122 (IL-2 Rβ)-PE, and analyzed with flow cytometry. One representative result from 3 independent experiments is shown. No B and T lymphocytes or NK cells were observed in the spleen of Hairless R/J mice

The generated Hairless R/J mice survived and reproduced well under specific pathogen-free conditions. Both heterozygous and homozygous Hairless R/J mice were born, and the first hairs were arising in the same condition. Heterozygous Hairless R/J mice gradually lost their hair after 4 weeks, as expected (Fig. 1A).

To confirm the expected immune phenotype of Hairless R/J mice, splenocytes were isolated and stained with anti-mouse CD3 (T cell marker)-Pacific blue and CD19 (B cell marker)-PE, and anti-mouse DX-5 (pan-NK marker)-APC and CD122 (IL-2 Rβ)-PE, and subjected to flow cytometric analysis. As shown in Fig. 1B, Hairless R/J mice showed no T and B lymphocytes and no NK cells, whereas Hairless mice showed CD3^+^ mature T cells, CD20^+^ B cells, and DX5^+^CD122^+^ NK cells.

### Permeability of fluorescence caused by the thin skin thickness of Hairless R/J mice compared with Nude R/J mice

**Fig. 2:**
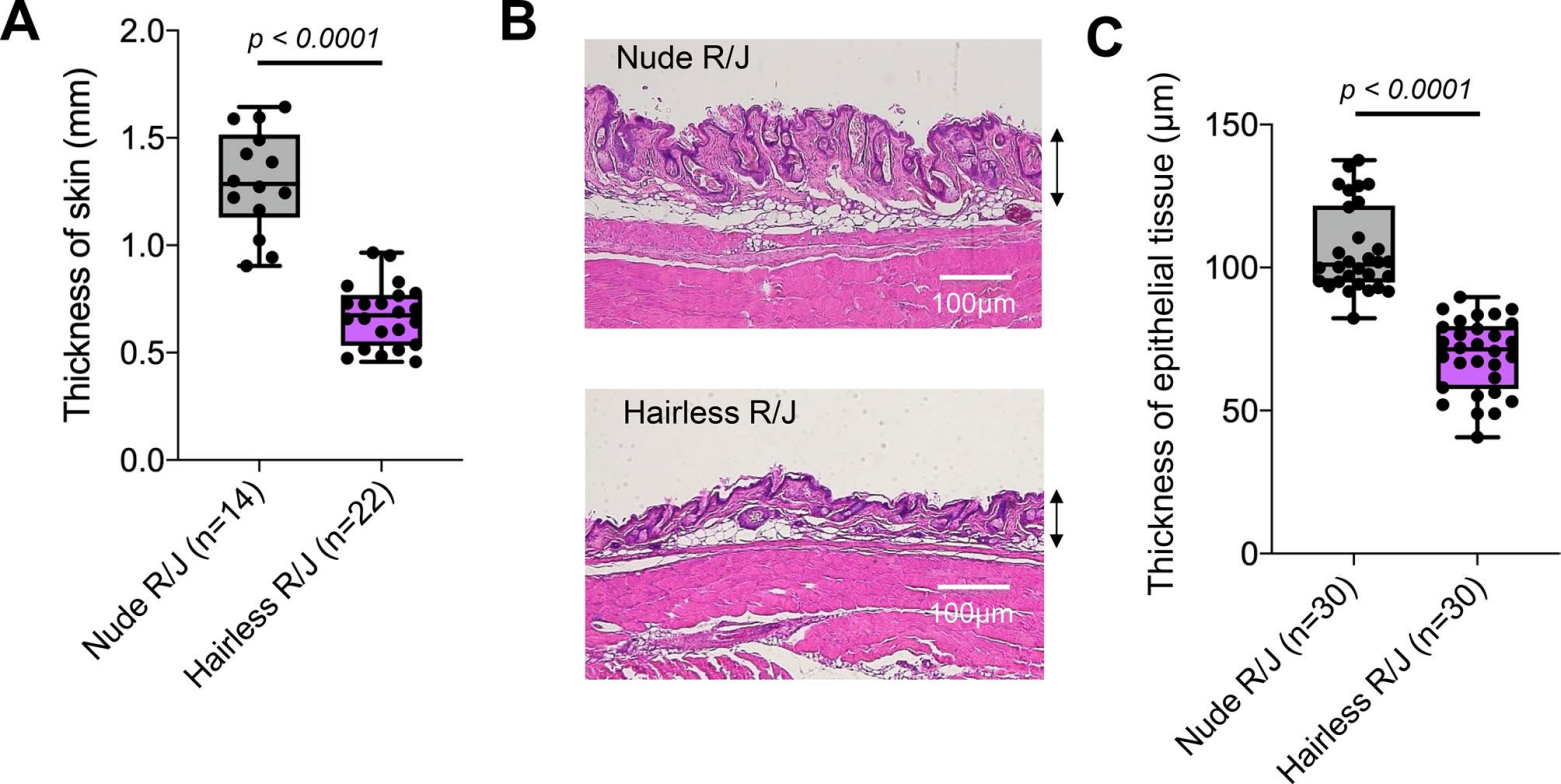
Differences in skin thickness between Nude R/J mice and Hairless R/J mice. (**A**) Skin thickness was measured using electric calipers at two locations per mouse, on the left and right sides of 8–10-week-old female mice. Statistical analysis of skin thickness was performed to compare the two mice. (**B**) Comparison of histological skin thickness by H & E staining. (**C**) Statistical analysis of epidermal thickness (as indicated by the arrows in Fig. 2B) was performed to compare the two mice

A direct comparison of skin thickness between Nude R/J mice and Hairless R/J mice was performed using electric calipers at two locations per mouse, left and right side (7 and 11 mice, respectively), revealing significantly thinner skin in the hairless phenotype (Nude R/J: 1.3 ± 0.24 mm, Hairless R/J: 0.67 ± 0.14 mm, *p* < 0.0001)(Fig. 2A). Skin histology specimens were prepared and stained with Hematoxylin & Eosin to compare the skin thickness of Nude and Hairless backgrounds. The thickness of the epidermal portion was measured at 3 parts each of 10 mice, as indicated by the arrow in Fig. 2B. As shown in Figs. 2B and 2C, the epidermis of Hairless R/J mice was thinner than that of Nude R/J mice (Nude R/J: 106.1 ± 15.2 mm, Hairless R/J: 69.2 ± 12.8 mm, *p* < 0.0001), indicating that fluorescence transparency may be higher in Hairless R/J mice than that of Nude R/J mice.

**Fig. 3:**
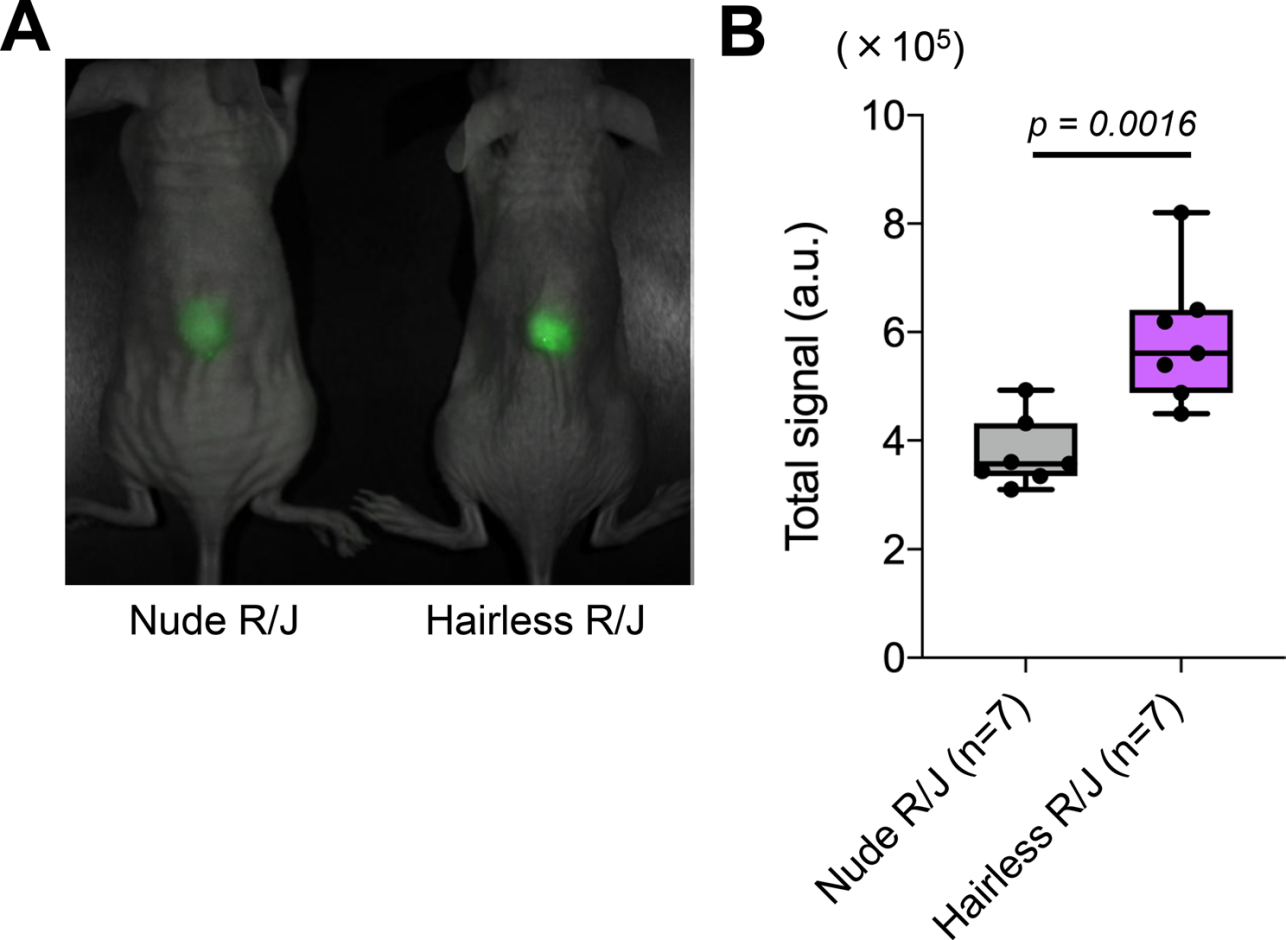
Differences in fluorescence intensity and the advantage of Hairless R/J mice in imaging analysis. (**A**) Comparison of fluorescence intensity between Nude R/J mice and Hairless R/J mice. Green fluorescently labeled beads were injected subcutaneously into 8–10 week-old female mice (*n* = 7), and the fluorescence intensity was analyzed using the Maestro in vivo fluorescence imager. (**B**) Statistical analysis of fluorescence intensity was performed to compare both mice

To confirm the fluorescence transparency, the Yellow-green fluorescence beads were subcutaneously administered to mice, and the fluorescence intensity was analyzed using the Maestro in vivo fluorescence imager. As shown in Fig. 3, the fluorescence intensity of the administered beads was brighter in Hairless R/J mice than that of Nude R/J mice (Nude R/J, 3.76 ± 0.64 × 10^5^ a.u.; Hairless R/J, 5.89 ± 1.23 × 10^5^ a.u.), suggesting that Hairless R/J mice were more useful than Nude R/J mice for fluorescence imaging.

### Visualization of non-invasive fluorescence of subcutaneous tumor cells

**Fig. 4:**
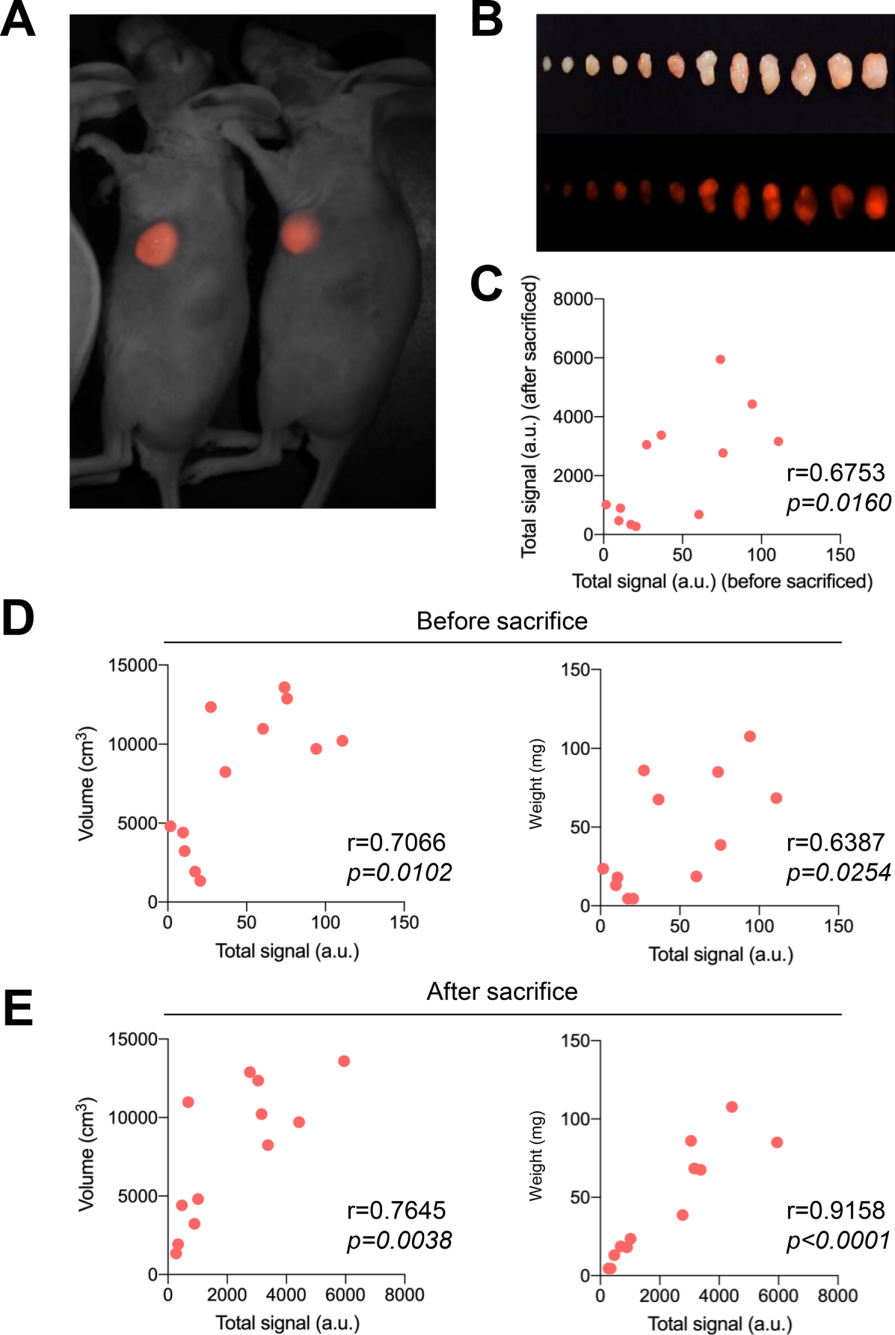
Visualization of mCherry-expressing tumor in Hairless R/J mice. (**A** and **B**) Direct red fluorescence of M213-mCherry tumor grown in the subcutaneous region of Hairless R/J mice (8–10-week-old female mice, *n* = 12) and visualized using a non-invasive fluorescence imaging system (Maestro). (**C**) Correlation of mCherry signals between outside (before sacrifice) and inside (after sacrifice). (**D** and **E**) Correlativity of tumor size and intensity of mCherry signals, or tumor weight and intensity of mCherry signals were compared at detection levels from outside or inside

The red-fluorescence labeled cholangiocarcinoma cell line, KKU-213A-mCherry, was subcutaneously transplanted into both flanks of the mice, and tumor fluorescence intensity in living mice was measured using Maestro. Then, the mice were sacrificed, tumors were excised, and and the fluorescence intensity, tumor size, and weight were measured. The fluorescence intensity of KKU-213A-mCherry detected in vivo and after sacrifice was compared to tumor size and weight (Figs. 4A and 4B). The in vivo fluorescence intensity was positively correlated with the tumor’s fluorescence intensity after sacrifice (Fig. 4C). Statistical analysis showed that both in vivo and post-sacrifice tumor fluorescence intensities positively correlated with tumor volume and weight (Figs. 4D and 4E). These findings suggest that in vivo fluorescence intensity in Hairless R/J mice correlates with tumor development and can be easily monitored over time in live animals.

## Discussion

In this study, we generated and characterized BALB/c Rag2/Jak3 double knockout hairless mice, completely deficient in lymphocytes and NK cells, using the hairless gene-deficient BALB/c strain (Hairless R/J mice). Since Hairless mice have thinner skin than Nude mice, they provide better visualization of tumor growth and progression, fluorescence imaging, and support effective development of human tumor cells.

Nude mice have been commonly used to study human tumor engraftment models because they have no hair and a T cell deficiency [14, 15]. Nude mice were discovered by Norman R Grist in 1962, and have been used for cancer research because of their lack of adaptive immune response due to a lack of thymus, and easy recognition of subcutaneous tumors by lack of body hair (Table 1) [4]. The responsible gene was identified the Forkhead box N1 (Foxn1) gene mutation on chromosome 11. SCID mice were first described by Bosma GC in 1983 and gained attention for their deficiencies in both B and T lymphocytes [16]. Hairless mice are another natural mutation with no body hair and have been used for skin research because their skin resembles human skin [17]; however, they are not used for human xenograft due to their standard immune system. Scid-Hairless Outbred (Crlj:SHO-*Prkdc* ^*scid*^*Hr* ^*hr*^ :SHO) mice were generated by Charles River in 2007 by intercrossing the SCID mice and Hairless mice [18–20]. SHO mice are homozygous for the SCID and Hairless (*Hr*^*hr*^), and exhibit the lymphocytes-deficient characteristics of SCID mice and hairless features. However, these mice retain NK cells, which are essential for tumor and xenograft rejection [6, 21, 22]. In addition, since both Nude and SCID mutations are natural mutations, they have lymphocyte leakage by aging and irradiation [23–25]. Furthermore, SCID mice are hypersensitive to ionized-radiation and anti-cancer drugs by the deficiency in DNA double-strand break repair [26]. We generated BALB/c Rag-2/Jak3 double-deficient (BRJ) mice to overcome these disadvantages [7]. BRJ mice lack Rag-2 and Jak3 by genetic knockout, resulting in a lack of B and T lymphocytes and NK cells, with no leakage. The BALB/c background can accept human cells and tissues due to SIRPα’s affinity for human CD47. As expected, BRJ mice efficiently accept human hematopoietic and tumor cells [7]. Based on these findings, we generated BALB/c Nude Rag-2/Jak3 double-deficient (Nude R/J) mice (7), which lack body hair and facilitate in vivo imaging [21, 22, 27]. Recently, Wei X et al. generated hairless, severe immunodeficient mice by deleting the Foxn1 gene using the CRISPR/Cas9 system in NOD/SCID/IL2rg^-/-^ Mice. Nude NOD/SCID/IL2rg^-/-^ (NSIN) mice exhibited improved capacity to graft both leukemic and solid tumor cells and facilitated the monitoring and in vivo imaging [28]. Both Hairless and Nude mice have been used as models of skin diseases and evaluation of dermatological drugs [17, 29], and these mice are also useful for in vivo imaging. Since Hairless R/J mice are derived from Hairless mice, they offer advantages for fluorescent imaging compared with Nude-based mice. However, the thickness of Hairless R/J mice’s skin is thinner than that of Nude R/J mice (Fig. 2), and as a result, the fluorescence intensity of Hairless R/J mice is stronger than that of Nude R/J mice (Figs. 3 and 4). In addition, skin-grafted human melanoma cells (Ihara) grow well and are easily observed from the outside (Supplementary Figure 1A), indicating that Hairless R/J mice are powerful tools for human research.

**Table 1:** Comparison of Nude/Hairless immunodeficient mouse strains

		Nude mice based					Hairless mice based				
mouse		Nude	Nude-J		Nude R/J	NSIN	Hairless	SCID Hairless Outbread	NOD/Scid Hairless	B-NDG hairless NOG-Hairless	Hairless R/J
Strain background		BALB/c	BALB/c		BALB/c	NOD	BALB/c	CB-17	NOD	NOD	**BALB/c**
Strain		BALB/c-*Foxn1*^*nu*^	BALB/c-*Jak3*^*tm*^*Foxn1*^*nu*^		BALB/c-*Rag-2*^*tm1*^*Jak3*^*tm1*^*Foxn1*^*nu*^	NOD.Cg-*Prkdc*^*scid*^ *Il2r*^*gtm1Sug*^ *Foxn1*^*nr*^	BALB/c-*Hr*^*hr*^	Crl:SHO*-Prkdc*^*scid*^*Hr*^*hr*^	NOD.Cg-*Prkdc*^*scid*^*Hr*^*hr*^	NOD.Cg-*Prkdc*^*scid*^ *Il2r*^*tm1*^*Hr*^*hr*^	**BALB/c-Rag-2**^***tm1***^*Jak3*^*tm1*^*Hr*^*hr*^
Immune cells	T cells	－	－		－	－	+	－	－	－	－
	B cells	+	－		－	－	+	－	－	－	－
	NK cells	+	－		－	－	+	+	±	－	－
Body hair		None	None		None	None	None	None	None	None	**None**
Skin		thick	thick		thick	thick	thin	thin	thin	thin	**thin**
Reference		4	24		8	31	9	21–23	a)	b), c)	

Reference: a) https://www.inotiv.com/research-model/nodcg-prkdcscidhrhr-ncrhsd, b) https://biocytogen.jp/immunodeficient-models/b-ndg-hairless-mice, c) https://www.ciem.or.jp/en/laboratory_animal/next-generation/pdf/next_NOG_mouse-2_Part25.pdf

Recently, NOD/Scid-based hairless mice have been established and are available from several animal companies, such as B-NDG hairless mice (BIOCYTOGEN Inc., Beijing, China), NOG-Hairless (LLEA Japan, Inc., Tokyo, Japan), and SHrN® hairless NOD.SCID mice (Inotiv, Inc., Lafayette, Indiana, USA) (Fig. 1), which have hairless and thin skin phenotypes; however, SHrN® hairless NOD.SCID mice have sparse, intermittent hair growth (https://www.inotiv.com/research-model/nodcg-prkdcscidhrhr-ncrhsd). We also established Hairless NOD/Scid/Jak3^null^ (NOJ) mice, which show sparse, intermittent hair growth (data not shown), suggesting that the NOD strain may have leakage of the Hairless gene. In addition, since NOD/Scid strains have a DNA-dependent protein kinase catalytic subunit (DNA-PKcs) mutation, they are sensitive to irradiation and DNA damage-inducing anticancer reagents compared with BALB/c strains [30, 31]. Since the radiation sensitivity of BALB/c mice is similar to that of humans [32, 33], indicating that the immunodeficient mice with a BALB/c background are more similar to humans in terms of irradiation and DNA-damaging drug sensitivities than NOD/Scid background mice, indicating that Hairless R/J mice can make more efficient human models.

## Conclusions

In this study, we generated Hairless R/J mice on a BALB/c background that are hairless, lymphocyte-deficient, and NK cell-deficient. Since Hairless R/J mice have thinner skin than Nude mice and are highly immunodeficient, they provide a valuable model for optical imaging in cancer research compared to conventional Nude mice. Their enhanced imaging properties make them a promising tool for evaluating tumor progression and therapeutic interventions in preclinical studies, particularly for humanized PDX models.

## Electronic supplementary material

Below is the link to the electronic supplementary material.

Supplementary material 1: Supplementally Figure 1. Engraftment of Ihara melanoma cell line into Hairless R/J mice. (A) Ihara cell line (1 × 106 cells/ mouse) was subcutaneously transplanted into Hairless R/J mice. (B) Three weeks after transplantation, mice were sacrificed, and immunohistochemistry of the tumor was performed by Masson-Fontana staining.

## Data Availability

The datasets used and/or analyzed in this study are available from the corresponding author upon reasonable request.

## Abbreviations

BRJ: BALB/c Rag-2/Jak3 double deficient mice
DMEM: Dulbecco’s Modified Eagle’s Medium
ECM: extracellular matrix
FBS: fetal bovine serum
Hairless R/J mice: Hairless Rag2/Jak3 double deficient mice
Hr: Hairless gene
KO: knockout
NOD: Non-obese diabetic
NSG: NOD-Scid IL2Rγ^null^
TDDS: transdermal drug delivery systems
