# Supplementary figures and images for: A novel hairless highly immunodeficient mice model optimized for in vivo imaging
Source: Lab Anim Res. 2026 Apr 7;42:11. doi: 10.1186/s42826-026-00275-9 (PMC13054980; doi:10.1186/s42826-026-00275-9)

## Slide 1
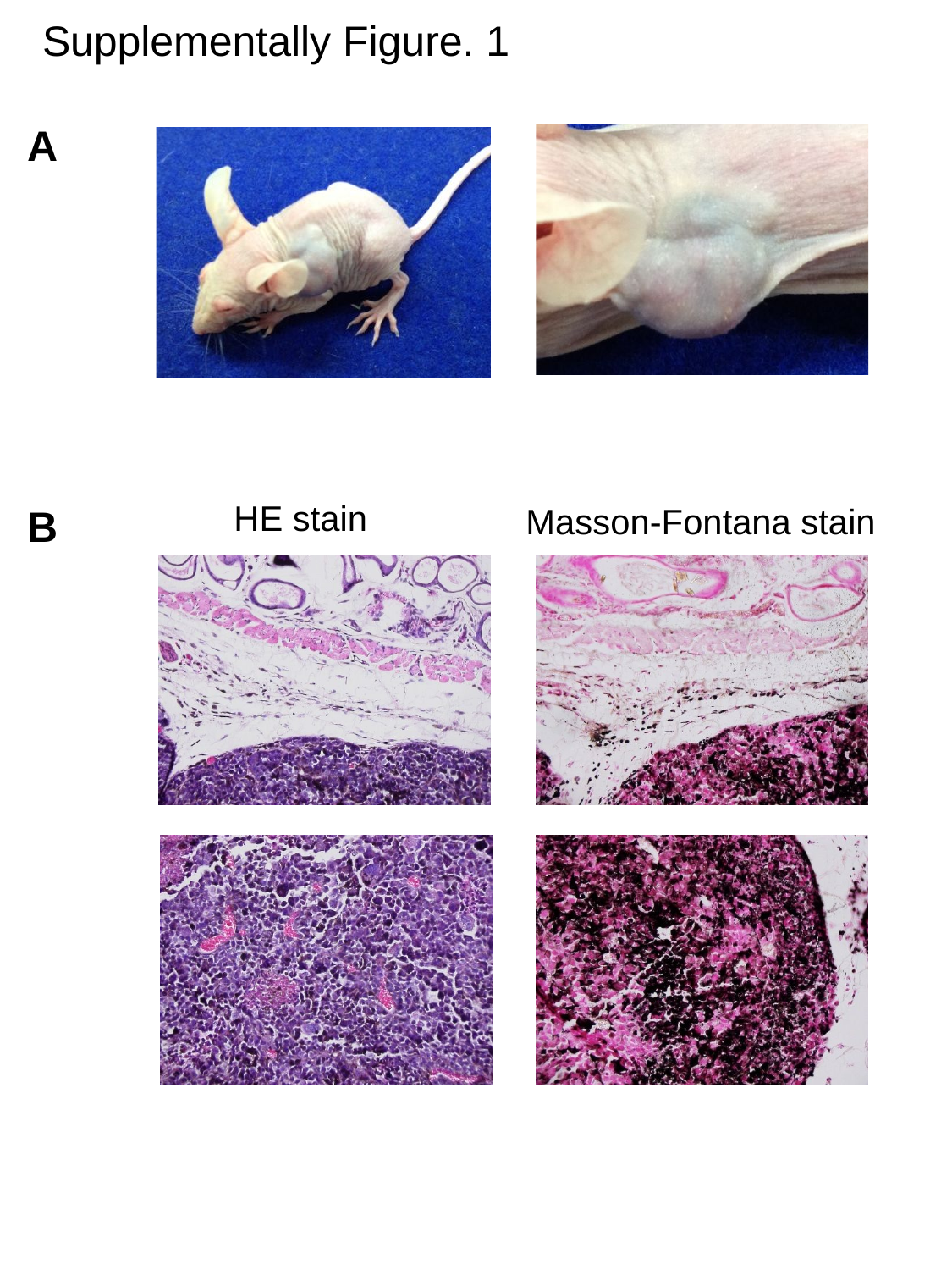

Supplementally Figure. 1
A
B
HE stain
Masson-Fontana stain

Supplement: Supplementary file 1 — Supplementary material 1: Supplementally Figure 1. Engraftment of Ihara melanoma cell line into Hairless R/J mice. (A) Ihara cell line (1 × 10^6^ cells/ mouse) was subcutaneously transplanted into Hairless R/J mice. (B) Three weeks after transplantation, mice were sacrificed, and immunohistochemistry of the tumor was performed by Masson-Fontana staining. [file 42826_2026_275_MOESM1_ESM.pptx]
